# Enhanced Bread Shelf Life Using Carboxymethyl Cellulose Composite Films with Combined Antibacterial Activity of Zinc Oxide and Cinnamon Essential Oil

**DOI:** 10.3390/foods15203641

**Published:** 2026-10-14

**Authors:** Ludmila Motelica, Ovidiu-Cristian Oprea, Roxana Doina Trusca, Anton Ficai, George Pelin, Denisa Ficai, Carmen Curutiu

**Affiliations:** 1 Research Institute of the University of Bucharest (ICUB), University of Bucharest, Spl. Independentei 91–95, 0500957 Bucharest, Romania; ludmila.motelica@upb.ro; 2 Research Center for Advanced Materials, Products and Processes, National University of Science and Technology POLITEHNICA Bucharest, Splaiul Independentei 313, 060042 Bucharest, Romania; 3 National Research Center for Micro and Nanomaterials, Faculty of Chemical Engineering and Biotechnologies, National University of Science and Technology POLITEHNICA Bucharest, Splaiul Independentei 313, 060042 Bucharest, Romania; 4 Academy of Romanian Scientists, Ilfov Street 3, 050044 Bucharest, Romania; 5 Faculty of Chemical Engineering and Biotechnologies, National University of Science and Technology POLITEHNICA Bucharest, Gh. Polizu 1–7, 011061 Bucharest, Romania; 6 INCAS—National Institute for Aerospace Research “Elie Carafoli”, Bd. Iuliu Maniu 220, 061126 Bucharest, Romania; 7 Faculty of Biology, University of Bucharest, 030018 Bucharest, Romania; carmen.curutiu@bio.unibuc.ro

**Keywords:** carboxymethyl cellulose, ZnO, biodegradable packaging, cinnamon oil, antibacterial, bread, pastry, shelf life

## Abstract

Cellulose is the most abundant natural polymer and is widely used in the packaging industry. One of the primary functions of food packaging is to protect food products from spoilage and extend their shelf life. Although cellulose lacks inherent antimicrobial activity, this limitation can be addressed through appropriate chemical or physical modifications to impart antimicrobial properties to cellulose-based materials. Nevertheless, cellulose represents a suitable matrix for the incorporation of various antimicrobial agents, enabling the development of active packaging materials. Moreover, the combination of multiple antimicrobial agents may result in combined effects, potentially providing broader and enhanced antimicrobial activity while reducing the overall amount of antimicrobial agents required. Herein, we report the preparation of a highly potent antimicrobial composite based on carboxymethyl cellulose (CMC) and zinc oxide (ZnO) nanoparticles (NPs) loaded with cinnamon essential oil (CEO). The composites were characterized using FTIR, UV–Vis, and fluorescence spectroscopy, scanning electron microscopy (SEM), and thermal analysis. Their mechanical and antibacterial properties, as well as potential biodegradability, were evaluated. The resulting films exhibited good barrier properties and enhanced mechanical performance. Although the composite films are composed of a food additive, spice extract, and ZnO NPs, all of which are generally recognized as safe (GRAS), the resulting innovative packaging exhibited strong antibacterial activity against *E. coli* and *S. aureus*, with inhibition zones reaching up to 38 mm. Moreover, no viable colonies of planktonic bacterial cells were detected after 4 h of incubation. In preliminary tests, no visible mold growth was observed on bread slices packaged in these films after 28 days of storage, whereas the control samples showed visible spoilage as early as day 7.

## 1. Introduction

Petroleum-based packaging materials are a growing concern from an ecological point of view, as they fragment into microplastics and contaminate the soil, waters and living organisms [1]. In the last decade, public pressure has led to growing interest in renewable, biodegradable materials that can be used in the food industry [2].

Cellulose is the most abundant natural biopolymer and can be obtained from a wide range of renewable sources, including wood, cotton, leaves, and agricultural residues [3]. Its linear structure consists of D-glucopyranose units linked by β-1,4-glycosidic bonds, with abundant hydroxyl groups that provide opportunities for chemical modification and the incorporation of functional additives. Cellulose is particularly attractive for food packaging because it combines renewability, biodegradability, biocompatibility, low cost, and excellent film-forming ability with good mechanical strength, transparency, and barrier properties against gases such as oxygen [4]. These characteristics distinguish cellulose from several other biopolymers and make it a versatile matrix for the development of sustainable packaging films. For example, although chitosan possesses intrinsic antimicrobial activity [5] and carrageenan has favorable film-forming and barrier properties [6], cellulose offers the advantages of exceptional availability and structural versatility, while its abundant hydroxyl groups facilitate the incorporation of nanoparticles and plant-derived bioactive compounds. However, native cellulose lacks inherent antimicrobial activity and is relatively hydrophilic, which can limit its resistance to water vapor. These limitations can be addressed through chemical modification or by incorporating functional agents, such as nanoparticles (NPs) and plant extracts, into the cellulose matrix. Therefore, the incorporation of suitable antimicrobial agents into cellulose-based films provides an effective strategy for developing active packaging materials capable of combining the inherent advantages of cellulose with enhanced antimicrobial functionality [7,8]. Carboxymethyl cellulose (CMC) has improved water dispersibility/film processability compared with native cellulose and is already widely investigated for food-packaging films [9,10,11].

Zinc oxide (ZnO) nanoparticles (NPs) are considered a good candidate due to their multiple antimicrobial mechanisms and because of their GRAS (Generally Recognized as Safe) classification by the US-FDA [12]. At the same time, the European Food Safety Authority (EFSA) considers that ZnO does not migrate in nanoform and has established that no toxic effects can be observed for zinc at levels up to 50 mg/person per day [13]. The combination of zinc ions with plant-derived antimicrobials has been reported to enhance the antimicrobial activity of clay-encapsulated carvacrol and thymol [14]. In addition, a synergistic antimicrobial effect has been reported for the combination of ZnO and carvacrol [15] or Zn-based MOF with marjoram essential oil [16].

Cinnamon essential oil (CEO) is extracted from all parts of cinnamon trees and contains up to 75% cinnamaldehyde [17]. It is considered a natural antimicrobial agent and a food preservative due to its strong antioxidant, antibacterial and antifungal activity, and is also classified as GRAS [18,19].

There are some previous reports on the combination ZnO/CEO in the food domain, such as milk-based beverages [20] or minced fish [21]. Our previous tests have indicated that the combination of ZnO and CEO exhibits a strong antimicrobial activity against bacterial strains like *Escherichia coli* (*E. coli*), *Staphylococcus aureus* (*S. aureus*), *Salmonella enterica* serovar Typhimurium (*S*. Typhimurium) and *Listeria monocytogenes* (*L. monocytogenes*) [22]. Nevertheless, only limited studies have investigated cellulose-based materials incorporating ZnO and cinnamon essential oil (CEO) for active packaging applications. For example, a ZnO–CEO emulsion for coating paper intended for the preservation of grey zucchini, demonstrating the potential of these antimicrobial agents when applied as a coating, was presented in [23]. In another study, ZnO and CEO were incorporated into a polyvinyl alcohol (PVA)-based formulation for the preservation of *Vigna unguiculata* [24]. CEO alone was incorporated into CMC, and the resulting composite was used for mutton preservation and exhibited strong antibacterial activity against *E. coli* and *S. aureus* [25]. However, these approaches differ from the present study in terms of the polymeric matrix and material configuration. The objective of this study was to develop and characterize cellulose-based composite films incorporating CMC, ZnO NPs and CEO for potential application as active food-packaging materials. The study aimed to investigate the influence of the ZnO NP/CEO ratio on the physicochemical, mechanical, barrier and antimicrobial properties of the films. Bread was selected as a model bakery product to evaluate the potential applicability of the developed films for the preservation of food products compatible with the characteristic fragrance of CEO.

## 2. Materials and Methods

### 2.1. Reagents

Zinc acetate dihydrate, Zn(CH_3_COO)_2_·2H_2_O, glycerol (C_3_H_8_O_3_), cinnamon bark essential oil (*Cinnamonum zeylanicum*; product number W229140), *n*-butanol, carboxymethyl cellulose, Tween 80 and citric acid were purchased from Sigma-Aldrich (Redox, Bucharest, Romania). ZnO NPs were prepared as previously reported in [12]. Briefly, 2.5 g Zn(CH_3_COO)_2_·2H_2_O was dissolved in 50 mL *n*-butanol and kept under reflux. After centrifugation and repeated washing with ethanol, ZnO NPs with a polyhedral shape and size of ~20 nm were obtained [26].

### 2.2. Preparation of Nanocomposite Film

A quantity of 5 g carboxymethyl cellulose (CMC) was dissolved under stirring in 100 mL of water. A volume of 2 mL of glycerol was added as a plasticizer. Separately, a suspension of ZnO NPs with CEO was prepared in 10 mL Tween 80 solution (concentration 10%) by ultrasonication [26]. The CMC, ZnO and CEO quantities together with the sample codifications are presented in Table 1. We have added 2 g citric acid as a reticulation agent in each case. The strategy was to have two series in which we increase the amount of ZnO (**1**; **2**; **3**) or the amount of cinnamon essential oil (**4**; **2**; **5**) separately.

The composition was poured into Petri dishes and left to dry in the oven at 40 °C for 24 h. The dried films were peeled carefully and were further stored in plastic zip-bags.

### 2.3. Characterization Techniques

#### 2.3.1. Fourier Transform Infrared Spectroscopy (FTIR) and Microscopy Measurements

FTIR spectroscopy was performed to identify the characteristic functional groups of the film components and to investigate possible chemical interactions between CMC, ZnO NPs and CEO. A Nicolet iS50 FTIR spectrometer (Thermo Fisher Scientific Inc., Waltham, MA, USA), equipped with an ATR module and a DTGS detector, was used to record the spectra in the wavenumber range 400 to 4000 cm^−1^. The measurement resolution is 4 cm^−1^, and each spectrum was obtained by averaging 32 scans [26].

FTIR microscopy was used to investigate the chemical composition and spatial distribution of the main functional groups within the composite films. FTIR maps were recorded with a Nicolet iS10MX FTIR microscope (Thermo Fisher Scientific Inc., Waltham, MA, USA), in the 4000–650 cm^−1^ range [26].

#### 2.3.2. Ultraviolet-Visible (UV-Vis) Spectroscopy

Ultraviolet-Visible (UV–Vis) spectroscopy was performed to evaluate the optical properties of the composite films. UV-Vis spectra were recorded in the range of 200–900 nm with a JASCO V560 spectrophotometer (Jasco Inc., Easton, PA, USA), in diffuse reflection mode. The spectrometer was equipped with a 60 mm integrating sphere and a film holder. Opacity values were calculated following the method described in [27], using Equation (1), where A_600_ represents the absorbance value at 600 nm, and x represents the film thickness in millimeters:

(1) Opacity = A_600_/x,

A higher opacity value indicates that the film is less transparent [26].

#### 2.3.3. Photoluminescence (PL) Spectra Measurements

Photoluminescence (PL) spectroscopy was used to investigate the fluorescence properties of the composite films and the effects of incorporating ZnO nanoparticles and CEO. The PL spectrum was recorded using a Perkin Elmer LS55 spectrometer (Waltham, MA, USA), using an excitation wavelength of 320 nm, a cutoff filter of 350 nm, a scanning speed of 200 nm × min^−1^ and 10 nm slits [26].

#### 2.3.4. Scanning Electron Microscopy (SEM)

The surface morphology and microstructure of the films were investigated by scanning electron microscopy (SEM) using a QUANTA INSPECT F50 microscope (FEI Company, Eindhoven, The Netherlands), equipped with a field-emission gun (FEG) electron source with a resolution of 1.2 nm and an energy-dispersive X-ray spectrometer (EDS) with an energy resolution of 133 eV at Mn Kα. The film samples were mounted onto metallic stubs using double-sided carbon adhesive tape and subsequently sputter-coated with a thin layer of gold [26].

#### 2.3.5. Simultaneous Thermal Analysis (STA) Measurements: Thermogravimetry (TG) Coupled with Differential Scanning Calorimetry (DSC)

Thermal behavior for each composite sample (~10 mg) was investigated using a STA 449C Jupiter, TG-DSC system from Netzsch (NETZSCH-Gerätebau GmbH, Selb, Germany), between 20 and 900 °C, with a heating rate of 10 °C/min, under an air flow of 50 mL/min [26].

#### 2.3.6. Water Vapor Permeability (WVP) Measurements

Water vapor permeability (WVP) was evaluated according to the method described in [7], using glass flacons (1.2 cm diameter) sealed with the test film. Each flacon contained 1.1 g anhydrous CaCl_2_. The sealed flacons were placed in a desiccator at 25 °C and 100% relative humidity (RH), generated by placing a vessel containing distilled water at the bottom of the desiccator to create a saturated water-vapor atmosphere. Each flacon was weighed at 2 h intervals over a total period of 12 h to quantify the mass of water vapor transmitted through the films. WVP values were then calculated by using Equation (2):

(2) $$\mathrm{WVP}=\;\frac{\left(M_{t}-M_{0}\right)}{∆t\times ∆P\times S}\times L,$$

where M_0_ and M_t_ are the flacons’ masses at the initial moment and at time t, L is the film thickness in meters, S is the film area at the flacons’ opening in m^2^, ΔP is the difference in water vapor pressure between the film sides at the temperature of 25 °C and a RH gradient of 100% (3169 Pa), and Δt is the time in seconds. The determinations were made in triplicates.

#### 2.3.7. Mechanical Properties Determination

The mechanical properties, such as tensile strength (TS), elongation at break (EB) and Young’s modulus, were determined using a Universal Testing Machine (Instron 5982, Norwood, MA, USA), equipped with a 5 kN load cell, consistent with guidelines stated in the ISO 527-1:2019 and ISO 527-3:2018 methods, using a specimen type 1B. The initial grip separation (gauge length) was set to 50 mm, and the sample was stretched at a crosshead speed of 10 mm/min. Three samples were measured for each film. The thickness of the samples was measured using a calibrated micrometer (Vogel Germany, Würzburg, Germany) in five random positions around the sample. TS (MPa) and EB (%) were calculated using Equations (3) and (4):

(3) $$\mathrm{TS}\;(\mathrm{MPa})=\;\frac{F_{\max}}{A},$$

(4) $$\mathrm{EB}\;(\%)=\frac{L_{2}-L_{1}}{L_{1}}\times 100,$$

where F_max_, A, L_1_ and L_2_ are the maximum load (N), initial cross-sectional area (m^2^), initial and final length of the sample. The Young’s modulus (MPa) values were determined from the slope of the elastic region within the stress-strain curves.

#### 2.3.8. Antimicrobial Qualitative Assessment

For antimicrobial activity, two reference bacterial strains were used: one Gram-positive strain—*Staphylococcus aureus* ATCC 25923 (*S. aureus*) and one Gram-negative strain—*Escherichia coli* ATCC 25922 (*E. coli*). For all microbiological analyses, the films, cut as 6 mm disks, were sterilized by UV for 20 min, before use. The assay was based on a modified Kirby–Bauer disk—diffusion method. From 24 h culture on nutrient agar, bacterial colonies were suspended in sterile physiological saline (SPS) to 0.5 McFarland microbial suspensions (1.5–3 × 10^8^ CFU/mL). The bacterial suspension was then spread over the surface of the nutrient medium using a sterile swab. Subsequently, the UV-sterilized samples were placed on the inoculated agar surface and plates were incubated for 18–24 h at 37 °C. The bacterial growth inhibition zones surrounding each sample were measured, and results were described as the diameter of the inhibition zone expressed in mm [26].

#### 2.3.9. Quantitative Analysis of Antimicrobial Effect

The samples were utilized to evaluate their antibacterial effects against free-floating (planktonic) microbial cells [26]. Briefly, an individual sample was placed in each well of a 24-well sterile plate. An initial bacterial concentration of approximately 10^6^ CFU/mL was introduced into each well by adding 10 μL of a microbial suspension adjusted to 0.5 McFarland in sterile physiological saline to 1 mL of nutrient broth. The prepared 24-well plates were incubated at 37 °C for 2, 4, 8, and 24 h. At each time point, serial tenfold dilutions of the corresponding samples were prepared, and 10 μL of each dilution was inoculated in triplicate onto nutrient agar plates for the enumeration of viable bacteria. Following incubation, the colonies were counted, and the arithmetic mean of the three replicate measurements was calculated and expressed as CFU/mL. When no visible colonies were detected, the result was reported as below the detection limit (BDL), <100 CFU/mL.

CFU/mL was calculated using the arithmetic mean of the colony counts from three replicate plates inoculated with 10 μL of sample, according to Equation (5):

(5) $$\mathrm{CFU}/\mathrm{mL}=\frac{\mathrm{Mean}\;\mathrm{colony}\;\mathrm{count}\;\times \;\mathrm{Dilution}\;\mathrm{factor}}{0.01}$$

where 0.01 mL corresponds to the inoculated volume of 10 μL.

#### 2.3.10. Preliminary Evaluation of Packaging Films for Bread Preservation

The effectiveness of the developed composite films in preserving bread was preliminarily evaluated by monitoring the visual appearance of bread specimens during storage. Bread samples (Bunatarie traditional farmhouse-style bread formulated without preservatives, purchased from a local market) were cut into cuboid specimens measuring 2 cm × 2 cm × 0.5 cm (length × width × thickness) and individually packaged in polyethylene (PE), neat CMC_0 film or the obtained CMC-based composite films. Each bread specimen was wrapped and secured by folding the film edges twice, without hermetically sealing the package. The packaged bread samples were stored under ambient laboratory conditions (approximately 25 °C and 30% relative humidity). The samples were visually inspected at predetermined time intervals for the appearance and development of visible mold growth. Representative photographs were taken throughout the storage period to document changes in the visual appearance of the bread samples. The experiment was performed in triplicate.

#### 2.3.11. Degradation Under Soil Burial Conditions

The degradation behavior of the composite films was evaluated using a soil burial test. Film specimens were cut into square pieces of approximately 2 × 2 cm, dried at 40 °C to constant weight, and accurately weighed to determine their initial dry mass (m_i_). The specimens were subsequently buried in natural soil at a depth of approximately 5 cm. The soil was maintained under controlled moisture conditions throughout the experiment to support microbial activity while preventing excessive waterlogging. The visual appearance of the films was recorded throughout the experiment to assess changes in color, integrity, cracking, fragmentation, and overall disintegration.

#### 2.3.12. Statistical Analysis

The obtained results were statistically analyzed using the analysis of variance (one-way ANOVA; with three replicates, n = 3) performed with Microsoft Excel 2016 (Microsoft Corp., Redmond, WA, USA), with the XLSTAT 2020.5.1 add-on installed. The Shapiro–Wilk test was used to check the normal distribution of the data; Levene’s test was used to assess the homoscedasticity of the residuals; the results were compared by Tukey’s (HSD) test so that the pairs of films that differed in terms of statistical significance were revealed (where *p* < 0.05).

## 3. Results and Discussion

### 3.1. FTIR Spectroscopy and Microscopy Results

**Figure 1:**
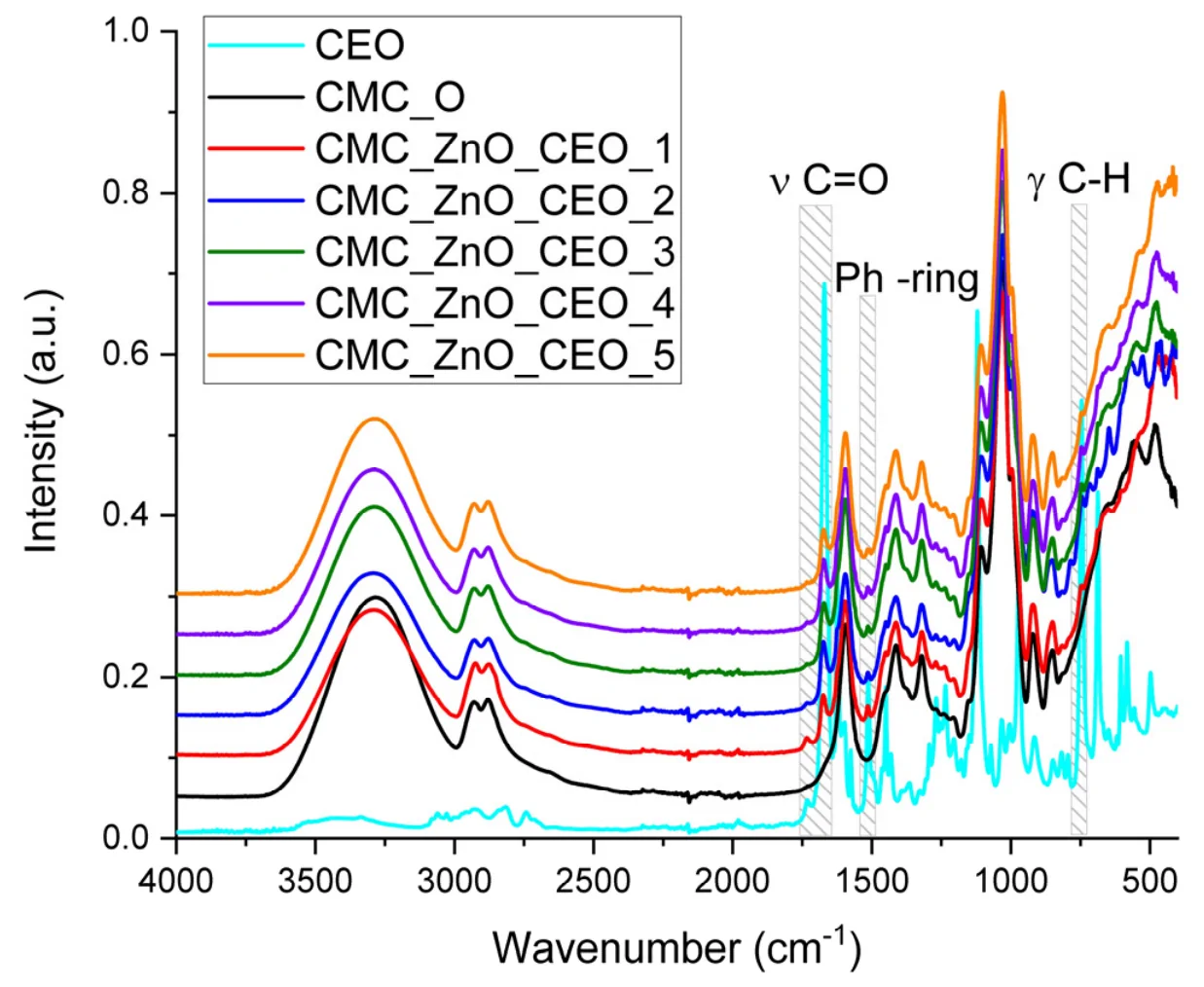
FTIR spectra for CMC_0 and CMC_ZnO_CEO_1–CMC_ZnO_CEO_5 composite films.

The presence of certain functional groups and the possible interactions between components of the nanocomposite films can be identified by using Fourier transform infrared spectroscopy (FTIR). The FTIR analysis of the CEO and films highlights several key characteristic absorption bands that define the components’ chemical structure (Figure 1). A broad band at 3288 cm^−1^ is attributed to the stretching vibrations of hydroxyl groups, indicating intermolecular and intramolecular hydrogen bonding, both in CMC and in CEO components like cinnamaldehyde, etc. [28,29]. The double peak from 2932 and 2877 cm^−1^ corresponds to C-H stretching vibrations (asymmetrical and symmetrical) from the CH_2_ moiety [7]. At the same time, the CEO FTIR spectrum presents specific peaks at 2744 cm^−1^ corresponding to the C-H stretching vibration in aldehydes, and 3023–3060 cm^−1^ generated by the unsaturated carbon—hydrogen bonds (cinnamaldehyde) [22].

The 1678 cm^−1^ peak observed in CEO and composite films, but not in the CMC_0 sample, corresponds to the C=O stretching vibration of cinnamaldehyde. The aldehyde usually appears at ~1725 cm^−1^ (very small intensity), but in cinnamaldehyde the C=O group is conjugated with the alkene and aromatic ring, which lowers the wavenumber to 1678 cm^−1^ [30]. The presence of the aromatic compounds in the composite films is also confirmed by the presence of the small peak at 1509 cm^−1^ originating from the aromatic ring skeletal stretching [31,32]. Additionally, the 741 cm^−1^ peak originates from out-of-plane C-H bending vibration from benzene rings [33]. These peaks confirm the incorporation of CEO into the final composite films.

The peaks at 1413 and 1596 cm^−1^ are assigned to the symmetric and asymmetric stretching vibrations of the -COO^−^ moiety, a fingerprint of CMC, which overlaps with 1601 cm^−1^ peak from CEO, corresponding to the aromatic -C=C-stretching vibration [26]. The CMC, like all polysaccharides, exhibits a very strong absorption band at 1029 cm^−1^ that is associated with the stretching vibrations of the carbon–oxygen (C-O) bonds in the ether linkages (C-O-C pyranose ring skeletal vibration) and in the polysaccharide ring [34,35,36].

The strong absorption band in the region of 410–420 cm^−1^ is assigned to the Zn-O stretching vibration [37].

**Figure 2:**
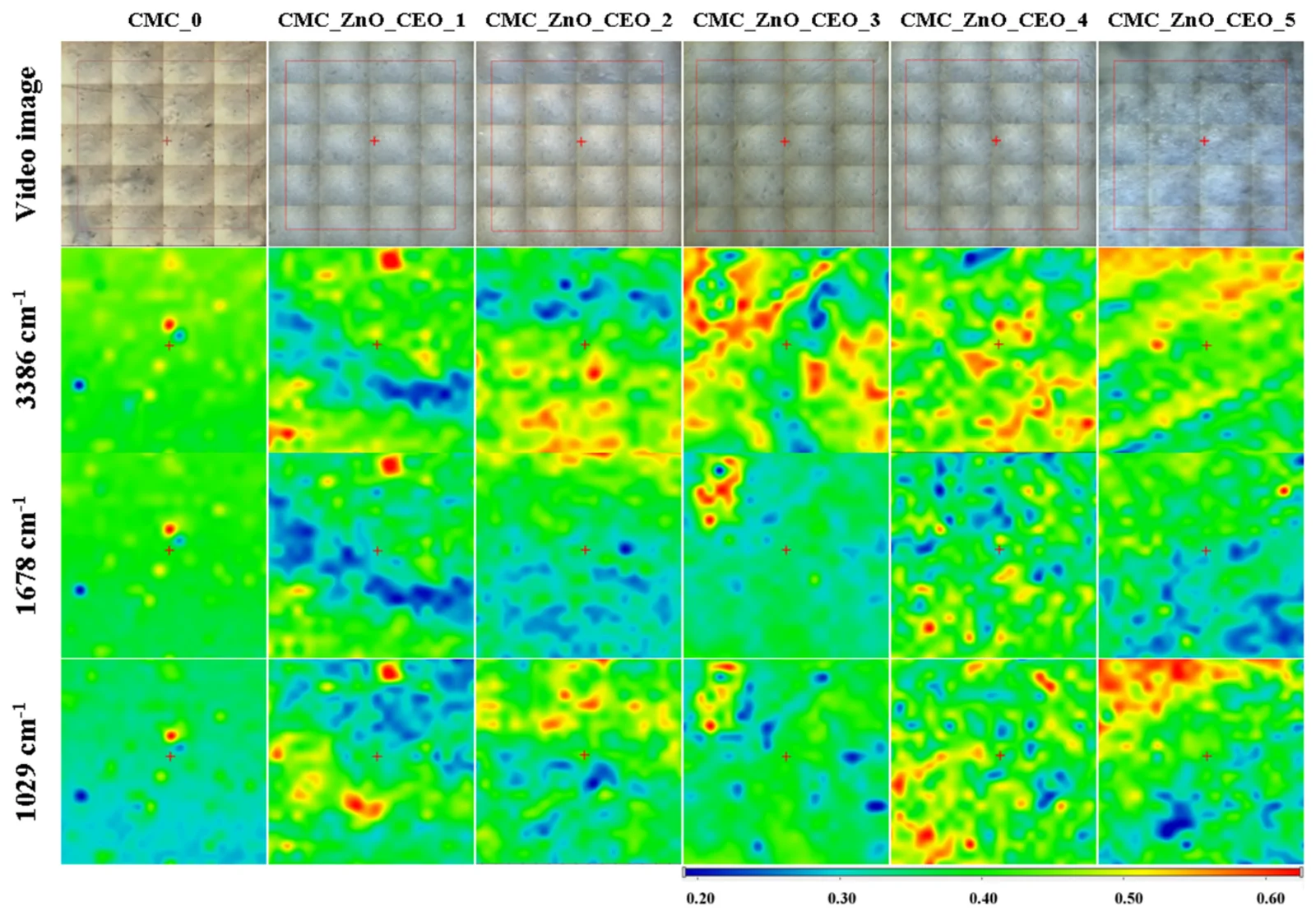
The FTIR maps for CMC_0 and CMC_ZnO_CEO_1–CMC_ZnO_CEO-5 composite films at wavenumbers 3386 cm^−1^, 1678 cm^−1^ and 1029 cm^−1^; red areas indicate the highest absorbance, while blue areas correspond to the lowest absorbance.

FTIR microscopy was used to assess the spatial homogeneity of the films by examining the distribution of characteristic infrared bands associated with the chemical components of the composite. Particular attention was given to the spatial distribution of bands assigned to CMC and CEO and to possible variations in chemical composition across the analyzed film areas. The FTIR microscopy maps (Figure 2) were collected at 3386 cm^−1^, corresponding mainly to O–H stretching vibrations of CMC and CEO; 1678 cm^−1^, associated with the C=O stretching vibration of cinnamaldehyde in CEO; and 1029 cm^−1^, assigned to C–O vibrations in CMC. The optical images show relatively uniform surfaces, with no visible evidence of phase segregation. The FTIR map at 1029 cm^−1^ exhibits only minor variations in absorbance, which may be related to small differences in film thickness or local variations in the concentration of CMC. The relatively homogeneous distribution of the bands at 3386 and 1678 cm^−1^ indicates a spatially uniform distribution of the corresponding O–H- and C=O-containing chemical groups, respectively, while the map at 1029 cm^−1^ indicates a relatively homogeneous distribution of the CMC-related C–O groups. These results support the overall chemical homogeneity of the analyzed film areas and suggest a relatively uniform distribution of CEO within the CMC matrix. However, the FTIR maps do not directly demonstrate the spatial distribution of ZnO nanoparticles; therefore, the absence of pronounced variations in the selected FTIR bands should not be interpreted as direct evidence of homogeneous ZnO dispersion (which was evaluated using SEM-EDS elemental mapping in Section 3.3).

### 3.2. UV-Vis and PL Spectrometry

UV–Vis analysis focused on the absorbance spectra over the investigated wavelength range, including the position and intensity of the absorption bands. The objective was to assess the light absorption and optical barrier properties of the films and to determine the influence of ZnO nanoparticles and CEO incorporation on their UV–visible light absorption behavior. The transparency of the obtained films against a colored label can be observed in Table 2.

The increase in the CEO or ZnO amount leads to a higher opacity for the composite films, but the overall transparency permits the visualization of the covered label. The samples CMC_ZnO_CEO_3 with the highest amount of CEO and CMC_ZnO_CEO_5 with the highest amount of ZnO present the highest opacity value, as indicated by the calculated values in Table 2.

Overall, the samples can be considered quite transparent, in line with previously reported values [26,27] for polysaccharide-based composites with ZnO NPs and essential oils. While the presence of a higher concentration of ZnO NPs can act as a physical barrier to light, decreasing the transparency, the higher amount of CEO creates, in fact, multiple micro-droplets that act as dispersion points for incoming light.

**Figure 3:**
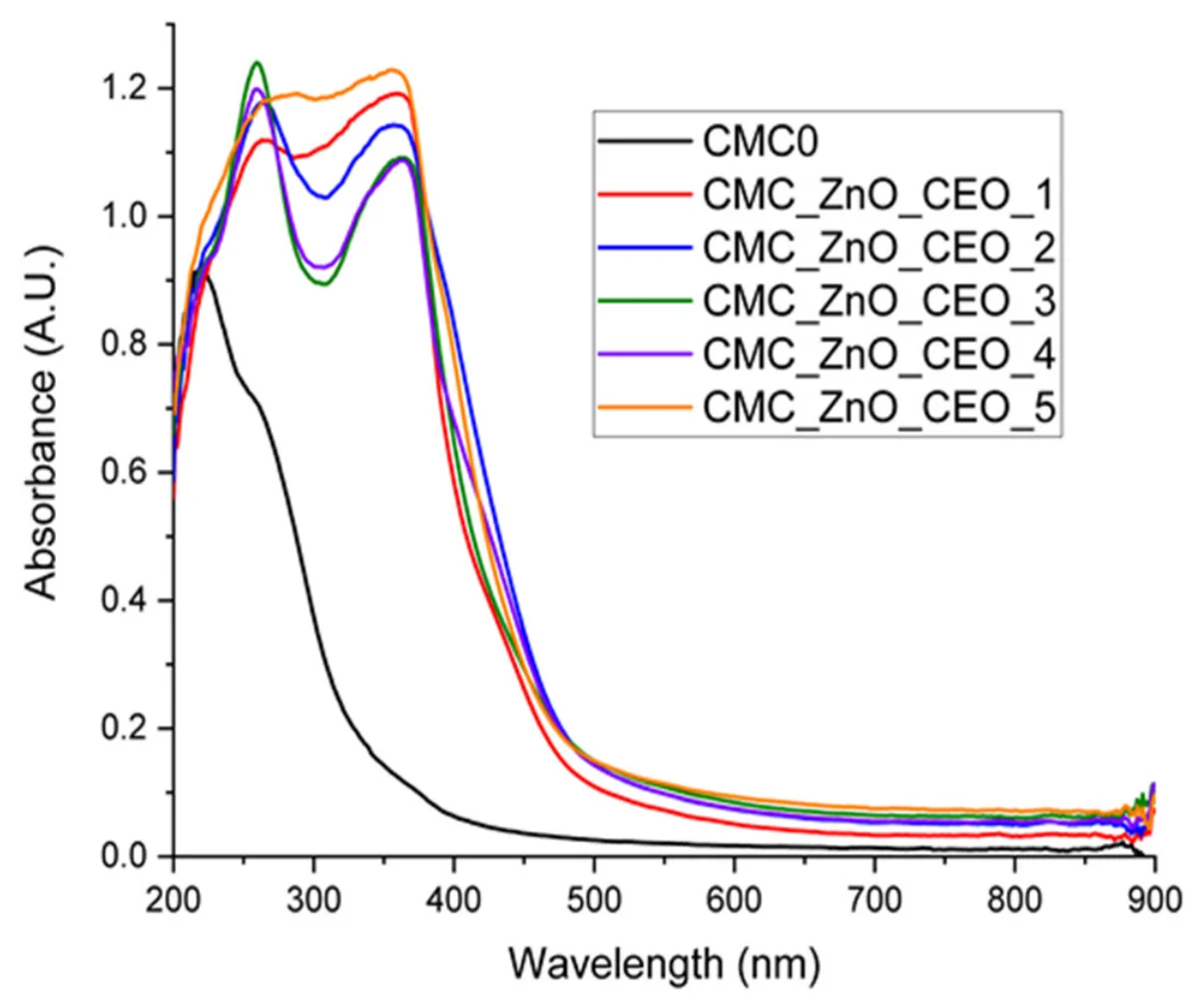
The UV-Vis spectra for CMC0 and CMC_ZnO_CEO_1–CMC_ZnO_CEO_5 composite films, with the UVA and UVB domains.

The transparency of the composite films in the visible domain is very important as customers demand to see the foodstuff. At the same time, blocking the UV radiation is equally important, as the high-energy photons are responsible for initiation of photo-oxidation reactions of lipids, vitamins, proteins and other nutrients. The obtained composite films are close to this ideal behavior, as indicated by the spectra presented in Figure 3.

While simple CMC_0 has a moderate blocking capacity for UV-C radiation, with the maximum around 220 nm, it does not block the UV-A and UV-B radiation. By contrast, all composite films exhibit very good UV absorption capacity, which partially extends into the violet-blue region of the visible domain.

The UV-Vis spectra exhibit a typical shape for the ZnO-based composites, with low absorbance in the visible domain and very good blocking properties in the UV domain. The ZnO NPs exhibit good absorption below 400 nm and are well known for their UV-blocking capacity. The ZnO-based composites preserve this ability in general, as it can be seen that the films presenting a large absorption band from just under 500 nm to 200 nm. The band has two distinct peaks at 380 and 290 nm, generated by ZnO and cinnamaldehyde (main compound from CEO). As expected, the intensity of the absorption peak at 290 nm, generated by cinnamaldehyde, increases from CMC_ZnO_CEO_1 to CMC_ZnO_CEO_3 as the incorporated CEO quantity increases. The absorption tail in the violet-blue region of the visible domain (400–500 nm) is responsible for the yellowish tint of the composite films.

**Figure 4:**
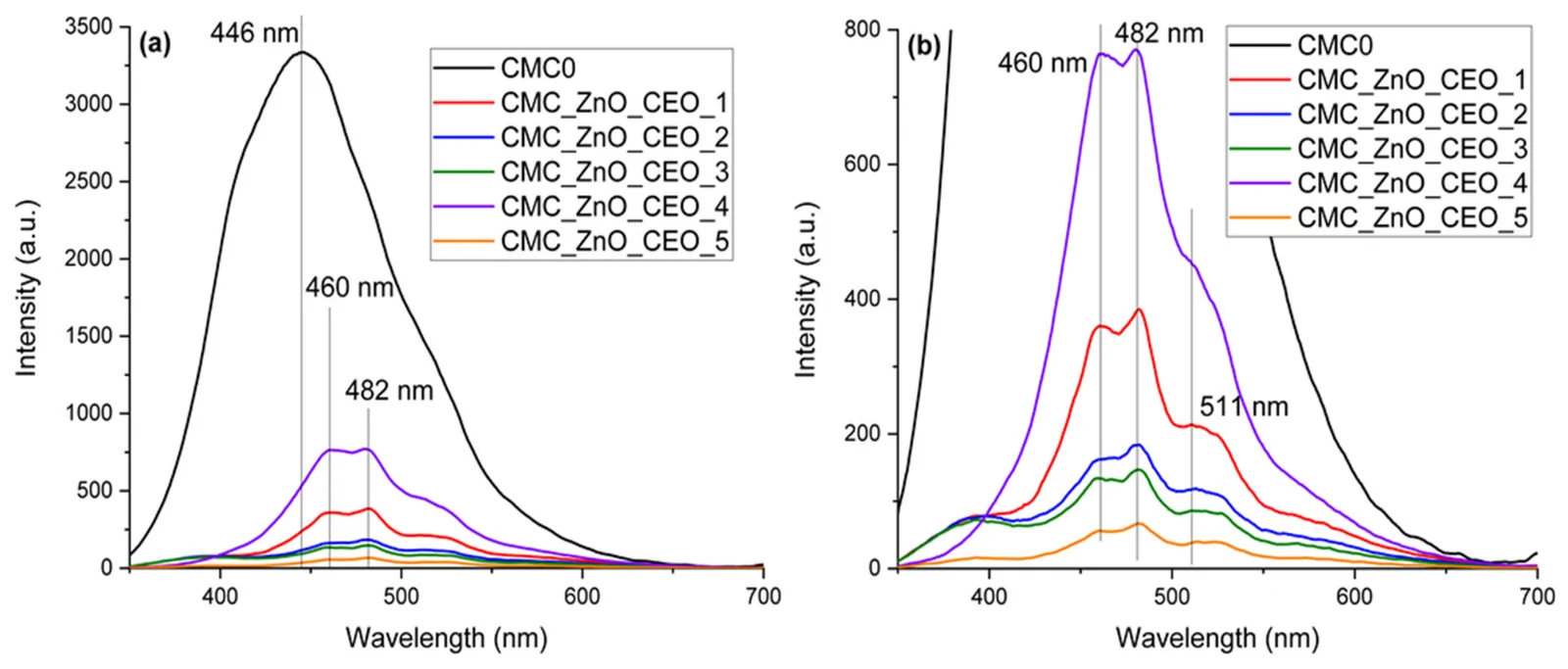
The PL spectra for CMC_0 and CMC_ZnO_CEO_1–CMC_ZnO_CEO_5 composite films (**a**); zoom-in detail (**b**).

The fluorescence emission spectra and corresponding emission intensity were evaluated, with the objective of identifying changes in the photoluminescent behavior of the films associated with their composition and possible interactions among the film constituents. The fluorescence spectra for the CMC_0 and composite films are presented in Figure 4. The cellulose derivatives, like other polysaccharides, exhibit a strong fluorescent emission peak, usually in the blue region of the visible spectrum, assigned to aggregation-induced emission (AIE) [26,38,39]. The observed fluorescence behavior differs from conventional fluorescence and may be associated with the aggregation of electron-rich functional groups resulting from the close packing of the polymer chains in the solid state. CMC contains numerous oxygen-containing groups with lone-pair electrons that may come into close proximity upon chain aggregation, potentially forming locally confined electron-rich domains. Such intermolecular interactions and restricted molecular motion may limit non-radiative relaxation pathways and consequently enhance emission in the solid state. Accordingly, the CMC_0 film exhibits a broad and intense emission band centered at approximately 446 nm.

Although ZnO NP are also known to exhibit strong fluorescence emission [12], the introduction of such particles in the composite films leads to a marked decrease in the overall emission intensity. The sample CMC_ZnO_CEO_4, with the lowest ZnO NPs quantity, exhibits the strongest fluorescence among the composite samples. The most probable explanation is that the addition of the ZnO NPs, but also CEO, alters the close packing of the polymer chains by intercalation among them, and therefore AIE is quenched. The ZnO NP acts as a crosslinking point for CMC, and therefore the surface of ZnO NPs is altered; the coating blocks the emission in the visible domain of ZnO (which is assigned in general to the surface defects) [40,41].

### 3.3. Scanning Electron Microscopy (SEM)

**Figure 5:**
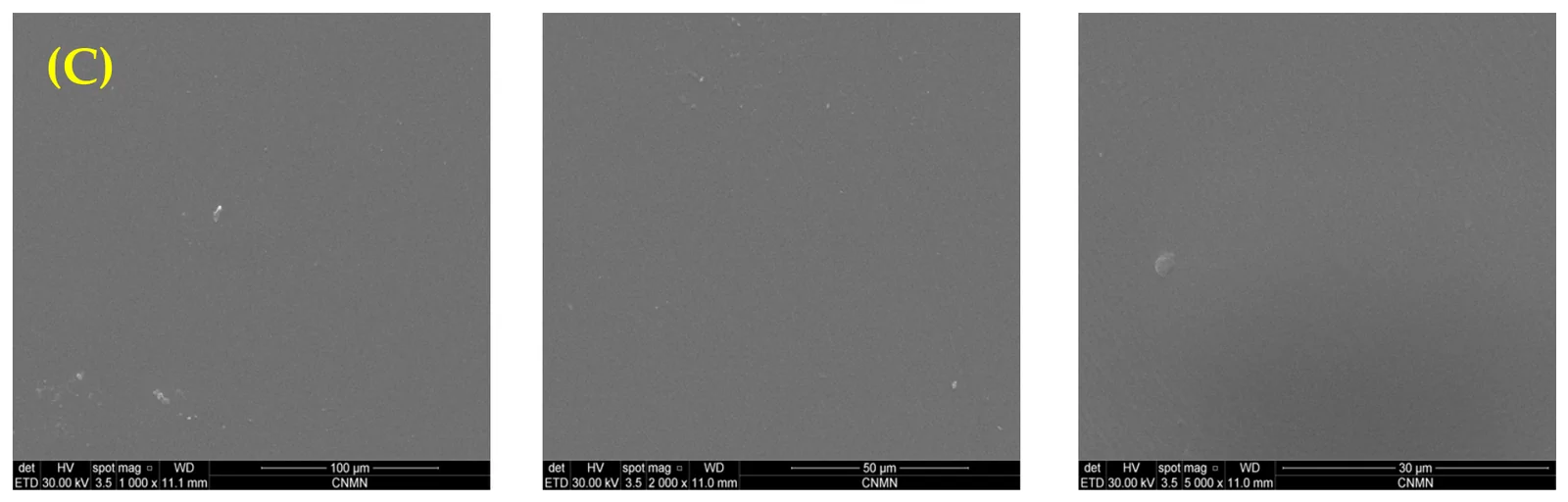

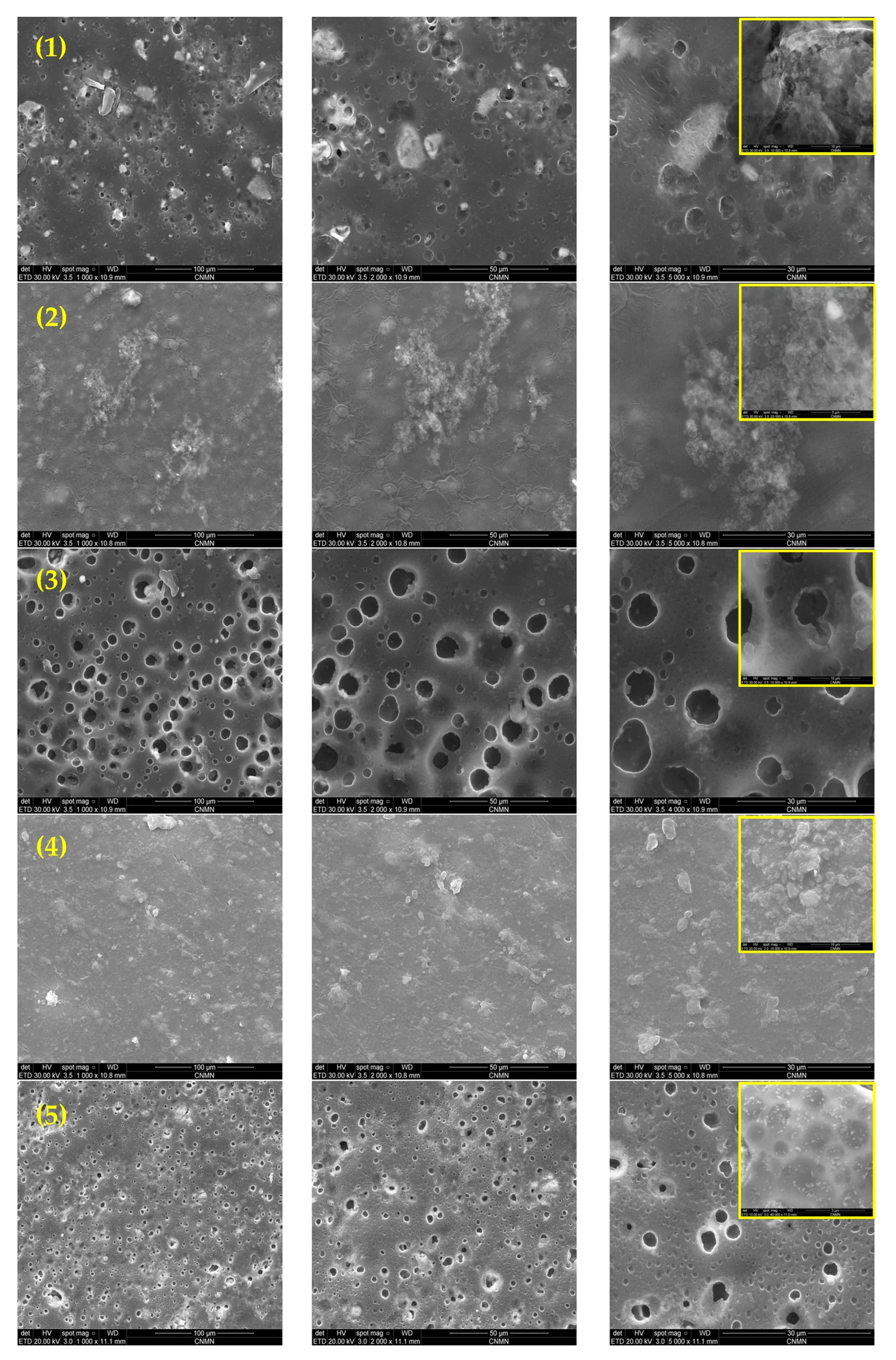
The SEM micrographs for the CMC_0 (C) and CMC_ZnO_CEO_1 (**1**)–CMC_ZnO_CEO_5 (**5**) composite films.

Scanning electron microscopy (SEM) was used to investigate the surface morphology of the CMC-based (Figure 5). Different magnifications were used for general view (1000×) and for detail evaluation (up to 10,000×). The SEM micrographs revealed differences in the surface morphology of the composite films depending on their formulation. Overall, the films exhibited relatively continuous matrices, although small agglomerated regions were observed in some samples, indicating that both ZnO NPs and CEO influenced the surface morphology of the films. The surface of the control film, CMC_0, was relatively smooth and homogeneous, as typically observed for polymeric films.

In contrast, the incorporation of ZnO NPs resulted in a rougher and more heterogeneous surface, with some detectable agglomerates. These agglomerations may be associated with the non-uniform dispersion of ZnO NPs within the CMC matrix and with possible interactions among ZnO, cellulose, and the CEO phase. Their presence suggests that complete dispersion of the inorganic phase was not achieved at all compositions. In addition, ZnO aggregates may generate microscale surface protrusions, contributing to the heterogeneous and rough appearance observed at higher SEM magnifications.

The incorporation of CEO further modified the surface morphology of the films, with the appearance of pores or void-like structures. The size and apparent density of these pores generally increased with increasing CEO content. The film containing the highest amount of CEO exhibited the most heterogeneous morphology, characterized by more clearly defined pores. These features may be associated with the presence and distribution of CEO microdroplets within the CMC matrix. During film drying, partial phase separation or migration of the CEO phase may occur, followed by the redistribution or loss of volatile oil components, leaving voids within the dried matrix [25]. The increased pore formation at higher CEO concentrations may therefore suggest reduced compatibility between the hydrophilic CMC matrix and the more hydrophobic CEO phase. Nevertheless, the SEM images alone cannot conclusively establish the mechanism responsible for pore formation, which may also be influenced by the drying conditions, the distribution of ZnO NPs, and the interactions among the different film components. Overall, the SEM observations indicate that increasing the ZnO and CEO contents progressively modifies the surface topography and microstructural organization of the CMC-based composite films.

**Figure 6:**
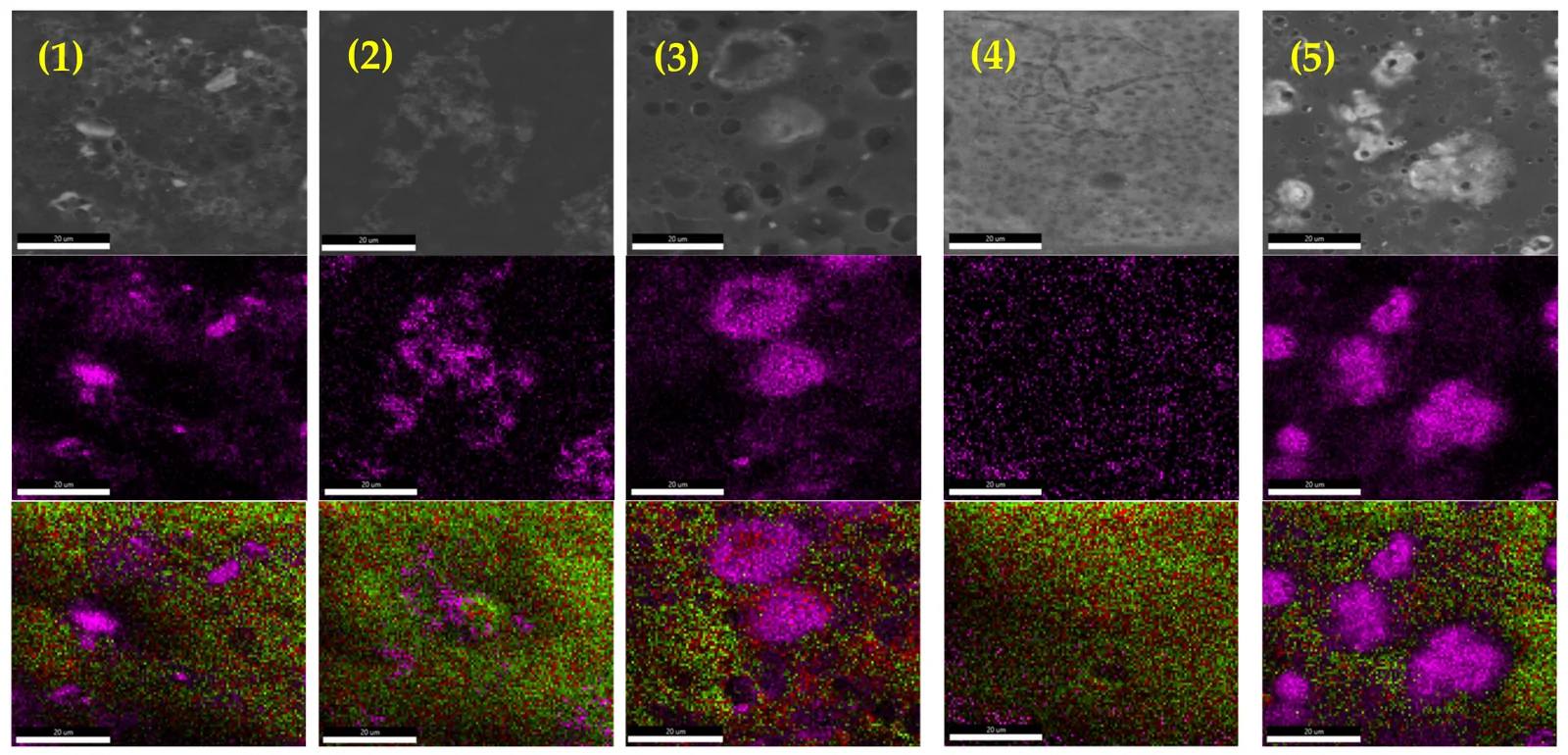
The EDX elemental maps for CMC_ZnO_CEO_1–CMC_ZnO_CEO_5 cellulose-based composite films (**Zn**—**purple**, **O**—**green**, **C**—**red**).

The elemental maps acquired by energy-dispersive X-ray (EDX) spectroscopy for zinc, carbon and oxygen are presented in Figure 6 and were used to assess the distribution of the ZnO NPs in the composite films. As can be seen, the CMC_ZnO_CEO_4 presents the highest homogeneity, with a smaller quantity of NPs leading to better dispersion into the polymer matrix. At the same time, by increasing the CEO amount, the micro droplets force the ZnO NPs to agglomerate in the remaining space, which generates a less homogeneous film.

### 3.4. Thermal Analysis

**Figure 7:**
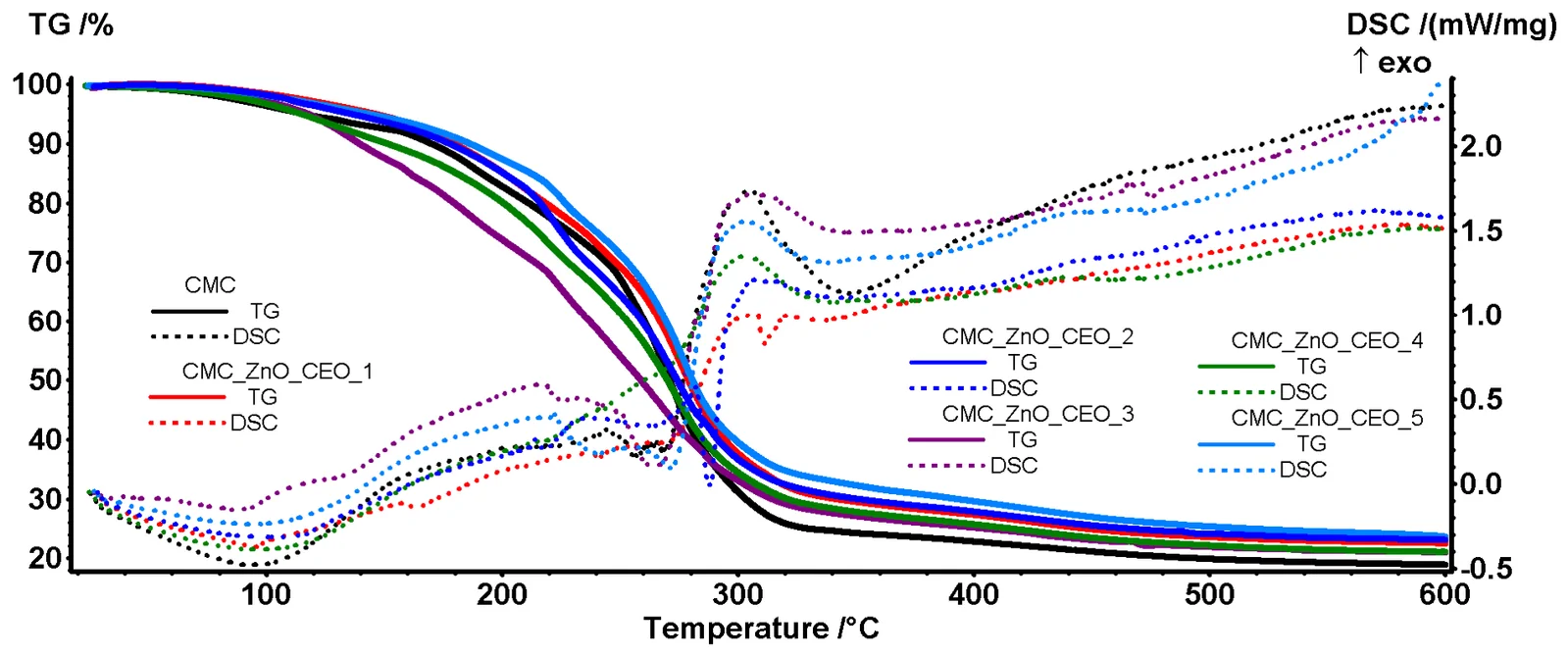
TG-DSC curves for CMC0 and CMC_ZnO_CEO_1–CMC_ZnO_CEO_5 composite films.

The thermal analysis results are presented in Figure 7 as thermogravimetric (TG) and differential scanning calorimetry (DSC) curves. The main numerical data are presented in Table 3, where T1%, T5% and T10% represent the temperature at which the sample lost the indicated percent of the initial mass.

The TG-DSC results revealed that the thermal behavior of the composite films was influenced by both the ZnO NPs and CEO contents. As expected, the neat CMC_0 film exhibited the lowest temperatures corresponding to the initial 1% mass loss (T1%), indicating the earlier release of residual moisture and other low-molecular-weight volatile species. In the composite films, the initial mass loss occurred at slightly higher temperatures, which may be attributed to interactions between the ZnO NPs surface and water molecules, as well as to the more tortuous pathway that water and other volatile species must follow through the composite matrix before reaching the film surface.

Among the composite samples, CMC_ZnO_CEO_4, containing the lowest amount of ZnO NPs, exhibited relatively low T_1% and T_5% values. This behavior may indicate weaker interactions between the inorganic phase and the volatile components, allowing their release at lower temperatures. Within the CMC_ZnO_CEO_4, CMC_ZnO_CEO_2, and CMC_ZnO_CEO_5 series, in which the ZnO NPs content increases, the progressive increase in T5% and T10% values suggests that the incorporation of a higher amount of ZnO NPs can delay the release of volatile components. This effect may result from interactions between the ZnO surface and water/volatile molecules and/or from the increased diffusion barrier created by the inorganic phase within the polymer matrix.

A different trend was observed when the CEO content was increased while maintaining a constant ZnO NP content, as in the CMC_ZnO_CEO_1, CMC_ZnO_CEO_2, and CMC_ZnO_CEO_3 series. In this case, the T1%, T5%, and T10% values progressively decreased with increasing CEO content. The lower onset temperatures are consistent with the higher proportion of volatile constituents introduced by the essential oil, which can be released from the matrix at relatively low temperatures. In particular, the pronounced decrease observed for CMC_ZnO_CEO_3 can be associated with its higher CEO content and, consequently, with a greater amount of volatile material available for release during the initial stages of heating.

Overall, the TG results suggest that the incorporation of ZnO NPs tends to delay the release of water and other low-molecular-weight volatile components, thereby increasing the apparent thermal stability of the films in the low-temperature region. In contrast, increasing the CEO content promotes mass loss at lower temperatures because of the volatile nature of the essential-oil constituents. These effects demonstrate that the thermal behavior of the composite films depends on the balance between the inorganic phase, which can restrict the mobility or release of volatile species and the CEO phase, which introduces additional thermally labile components.

At temperatures above ~150 °C, the subsequent mass-loss stage can be mainly associated with the thermal degradation of the polysaccharide matrix, involving dehydration, depolymerization and decomposition of the backbone chains. The DTG curve of the CMC_0 film showed a maximum degradation rate at 268.8 °C, providing an indication of the principal degradation temperature of the neat CMC matrix. The thermal events observed at higher temperatures, particularly above 300 °C, can be attributed to the further degradation and oxidation of the residual carbonaceous material formed during the preceding decomposition stages. The DSC exothermic effects in this temperature region are therefore consistent with the oxidation of the remaining carbonaceous residue.

### 3.5. Water Vapor Permeability (WVP) Results

An important function of food packaging is to control moisture transfer between the packaged product and the surrounding environment. Excessive moisture loss may cause food products to become dry and harder, resulting in undesirable changes in their organoleptic properties. Conversely, excessive moisture uptake in dry food products can promote quality deterioration and spoilage. Therefore, water vapor permeability (WVP) is a critical property for evaluating the suitability of packaging materials for moisture-sensitive food products [42]. The CMC, like all hydrophilic polymers, has poor water barrier properties, as water molecules can permeate by a hydrogen-bond-mediated mechanism [43,44]. The obtained WVP values are presented in Table 4, with the pristine CMC_0 exhibiting the highest WVP value.

In the composite films, the incorporation of both ZnO NPs and CEO resulted in lower WVP values. The ZnO NPs can act as physical barriers to water-vapor diffusion, while the hydrophobic nature of CEO may further restrict water transport through the film matrix. In addition, ZnO NPs may act as cross-linking or physical interaction points for the CMC chains, restricting their mobility and contributing to a more compact and rigid polymer network [43,45]. These effects can increase the tortuosity of the diffusion pathway and consequently reduce water-vapor transport through the films [46].

The obtained WVP values support this mechanism, with statistically significant differences observed with increasing CEO content. The reduction in WVP may therefore be associated with the combined effects of the hydrophobic oil phase and the physical barrier provided by the ZnO NPs. However, within the series with increasing ZnO NPs content, a statistically significant difference was observed only for the sample containing the highest ZnO NPs concentration. This finding suggests a weaker concentration-dependent effect of ZnO and may also indicate that, at higher concentrations, NPs agglomeration limits the effective barrier contribution of the inorganic phase.

### 3.6. Mechanical Characterization of the Films

The mechanical properties for all the samples are presented in Table 4. The mechanical performance of the samples was assessed based on tensile strength (TS), Young’s modulus, and elongation at break (EB), which collectively characterize the strength, stiffness, and flexibility of the developed materials [47]. The presence of CEO decreases the inter-chain interactions and limits efficient stress transfer in the polymer matrix [10]. The CEO acts as a plasticizer, increasing the mobility of the polymer chains, mainly by reducing the hydrogen bonding, lowering TS and increasing EB [25,48,49]. On the other hand, the presence of ZnO NPs dispersed into the polymer matrix leads to an increase in TS and a lowering of EB. The inorganic NPs act as reinforcing fillers and cross-linking points for the polymer chains, forming hydrogen bonds between the NPs’ surface and CMC, allowing a more efficient stress transfer, while bearing a fraction of the applied load [46,50]. This mechanism restricts CMC chains’ mobility and generates a stiffer matrix. At the same time, local ZnO NPs agglomerations can act as a stress concentration from which cracks can initiate. Therefore, the presence of both CEO and ZnO NPs induces competing effects in the composite films and the determined values are the result of such a balance [45,51].

### 3.7. Antibacterial Activity Assay

**Figure 8:**
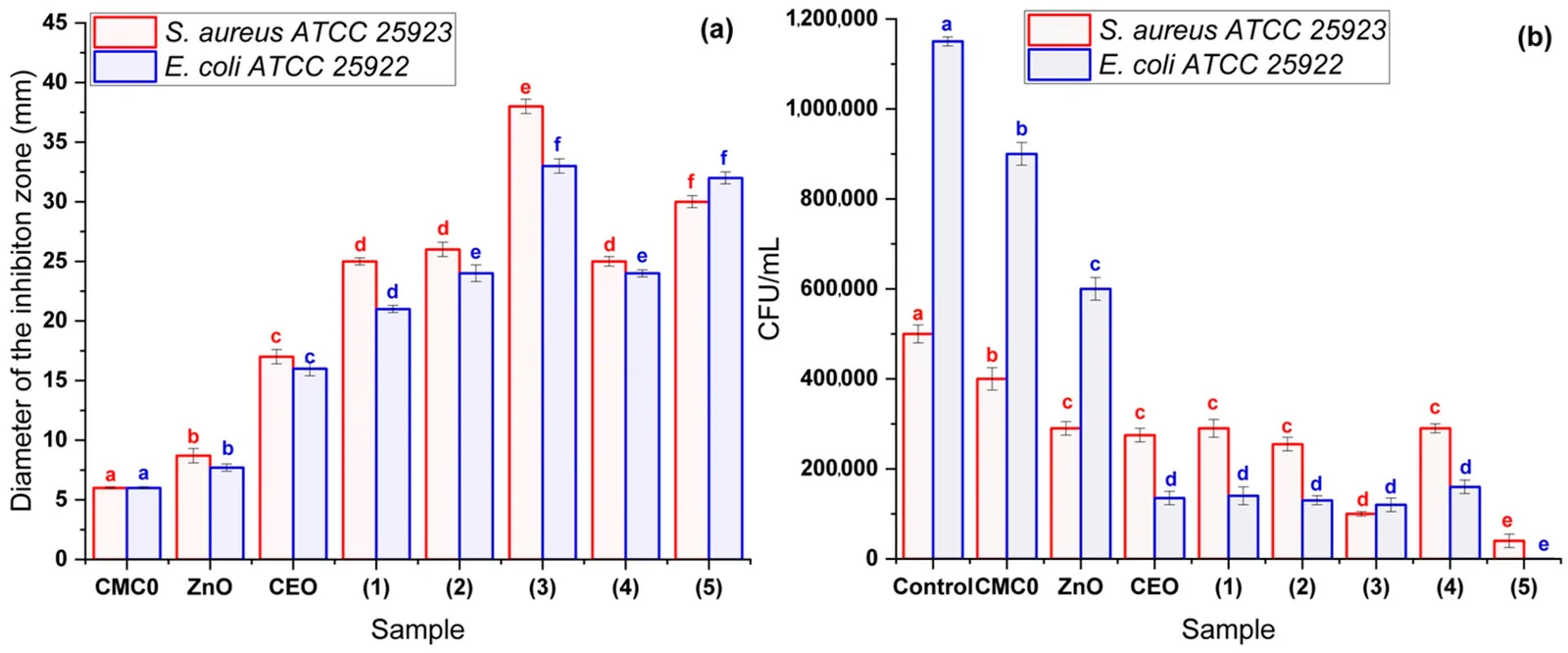
The diameter of the inhibition zone (**a**) and planktonic growth at 2 h (**b**) for CMC_0 and CMC_ZnO_CEO_1 (**1**)–CMC_ZnO_CEO_5 (**5**) composite films, against *S. aureus* ATCC 25923 (red) and *E. coli* ATCC 25922 (blue). Samples sharing the same letter of the same color are not significantly different (*p* > 0.05).

*E. coli* and *S. aureus* were chosen as model bacterial strains (Gram–negative and Gram–positive) for evaluating the antibacterial activity of the obtained composite films and assessing the capacity to prevent food contamination. Both *Escherichia coli* and *Staphylococcus aureus* are species frequently implicated in food contamination and can be responsible for causing foodborne illnesses. The results are presented in Figure 8, showing the diameter of the inhibition zone (Figure 8a) and colony-forming units per mL after two hours’ contact (Figure 8b).

Qualitative analysis showed that all samples exhibited strong antibacterial activity for both *E. coli* and *S. aureus* strains, with the diameter of the inhibition zone between 21 and 38 mm (Table S1), depending on ZnO and CEO content (Figure 8a). The CMC_ZnO_CEO_3 sample, with the highest CEO concentration, exhibited the largest inhibition zones, 33 mm for *E. coli* and 38 mm for *S. aureus*. The fact that *S. aureus* is more susceptible to CEO presence alone was already confirmed previously in the literature [22,52,53].

The main reason for this behavior could be the differences in bacterial cell-wall structure between Gram-positive and Gram-negative strains. The thick layer of the lipopolysaccharide outer membrane protects *E. coli* better compared with the *S. aureus’* single peptidoglycan layer structure [54]. For the series (4)-(2)-(5), in which the ZnO NPs amount increases, the samples with the highest ZnO content presented the largest inhibition zones, but the increase was smaller than the CEO corresponding one, with 32 mm for *E. coli* and 30 mm for *S. aureus*. This indicates that overall, *E. coli* is more susceptible to ZnO presence than *S. aureus* [12]. This phenomenon was observed before, as the inhibition zone for *S. aureus* shows signs of saturation while the inhibition zone of Gram-negative *E. coli* expands with increasing ZnO amount [55]. Also reported, the IC_100_ for *E. coli* was smaller than the value for *S. aureus,* confirming this behavior [56]. The antimicrobial mechanisms proposed for ZnO NPs are quite complex, and it is reported that the activity is also size- and morphology-dependent [57]. The main hypothesis is that ZnO is producing reactive oxygen species (ROS) that damage cellular structures. At the same time, the NPs tend to cluster at the interface with the cell’s membrane and can cause mechanical damage besides a localized increase in ROS concentration [58]. Such mechanical damage can include abrasion, puncture or even rupture of the membrane, leading to leakage of essential components like K^+^, RNA or proteins [59]. Lastly, ZnO NPs can be internalized and release Zn^2+^ ions inside or near the cell membrane. These ions interfere with the biochemical processes and pathways by binding essential components and nutrients of the cell [59].

Quantitative assay results for the viability of bacterial cells in contact with the composite films are presented in Table S2 and Figure 8b. The impairment of viability was clearly visible after 2 h for all samples; the one with the highest ZnO content, CMC_ZnO_CEO_5, exhibited the most potent action for both strains, with complete inhibition of growth in the case of *E. coli*, confirming the sensitivity of this strain to ZnO presence. Recently, it was also reported that ZnO NPs can downregulate the expression of the Ag43 surface protein, responsible for biofilm formation and bacterial adherence, indicating a novel antimicrobial pathway for ZnO NPs [60]. Finally, after 4, 8 and 24 h of contact, the results were below the detection limit for both *S. aureus* and *E. coli* strains, with no viable microbial cells being recovered on nutrient agar. The significant decrease in bacterial viability can be related to the release and presence of the bioactive compounds in the growth media. However, the antibacterial activity against *E. coli* and *S. aureus* was comparable, with only limited differences observed in both the inhibition zones and bacterial viability (Figure 8). Therefore, based on the present results, no clear distinction can be established regarding which of the two strains is more sensitive to the tested treatment. These findings suggest that the antibacterial effect was broadly similar against both Gram-negative *E. coli* and Gram-positive *S. aureus*.

CEO’s primary active component is cinnamaldehyde, which disrupts microbial cell walls, leading to cell death. There are multiple studies indicating that cinnamaldehyde inhibits biofilm formation by interfering with quorum-sensing pathways, especially by reducing virulence factor expression [61,62,63]. The lipophilic nature of cinnamaldehyde facilitates its interaction with and penetration into microbial cell membranes, resulting in membrane disruption, increased permeability, leakage of intracellular components, and ultimately cell damage [64]. In a previous study, cinnamaldehyde exhibited strong binding to the bacterial cell-division essential microbial proteins like FtsZ, forming hydrogen-bond interactions within its active site, which may contribute to the antibacterial and antibiofilm activity of CEO [65].

One possible explanation for the observed antibacterial activity is that CEO compromises the bacterial cell envelope, thereby increasing the susceptibility of bacterial cells to the multiple antimicrobial mechanisms of ZnO NPs. Such a proposed mechanism is consistent with previous studies reporting enhanced antimicrobial activity when ZnO is combined with cinnamaldehyde or essential oils. Ding et al. [66] demonstrated a synergistic antibacterial effect between ZnO and cinnamaldehyde/tea polyphenols, accompanied by pronounced deformation and folding of the bacterial cell membrane, increased membrane permeability, leakage of intracellular components and disruption of protein expression. Although cinnamaldehyde was used rather than whole CEO in that study, it is a major bioactive constituent of CEO and therefore provides mechanistic support for the proposed interaction. More directly, Jose et al. [24] reported enhanced antimicrobial activity of CEO-containing PVA films incorporating ZnO NPs and nanocellulose and observed morphological alterations in treated *S. aureus* and *E. coli*. Their results also showed that incorporation of ZnO increased the release of cinnamaldehyde from the CEO-containing matrix, suggesting that ZnO may enhance the availability of CEO antimicrobial constituents. Furthermore, Motelica et al. [22] demonstrated synergistic antibacterial activity for ZnO NPs combined with several essential oils, including CEO. The authors proposed that essential oils may sensitize bacterial cells by altering cellular homeostasis and membrane properties, thereby facilitating ZnO-mediated membrane damage and the interaction of Zn^2+^ ions with intracellular proteins and enzymes. Collectively, these findings support the hypothesis that CEO-induced perturbation of the bacterial envelope may increase cellular susceptibility to the membrane-disruptive, ion-mediated and oxidative antimicrobial effects of ZnO NPs.

As a future research direction, the present study will be expanded to investigate the molecular and cellular mechanisms underlying the antimicrobial activity. Moreover, additional microbial strains will be investigated to provide a more comprehensive evaluation of the antimicrobial spectrum, and fungal strains will also be included, considering that filamentous fungi are among the responsible species for bread spoilage.

### 3.8. Evaluation of Packaging Films for Bread Preservation

The use of essential oils in active packaging requires consideration of their compatibility with the intended food product. For example, oregano essential oil may be particularly suitable for meat products, thyme essential oil for cheese and cinnamon essential oil for bakery and pastry products. Accordingly, the performance of the developed packaging films was evaluated using bread as a model bakery product. The antimicrobial performance of the films was assessed by monitoring the development of visible mold growth on bread samples packaged with the composite films, CMC_0 and PE as a control. Surface mold growth is one of the major factors contributing to the reduction of the shelf life of bakery products [67].

**Figure 9:**
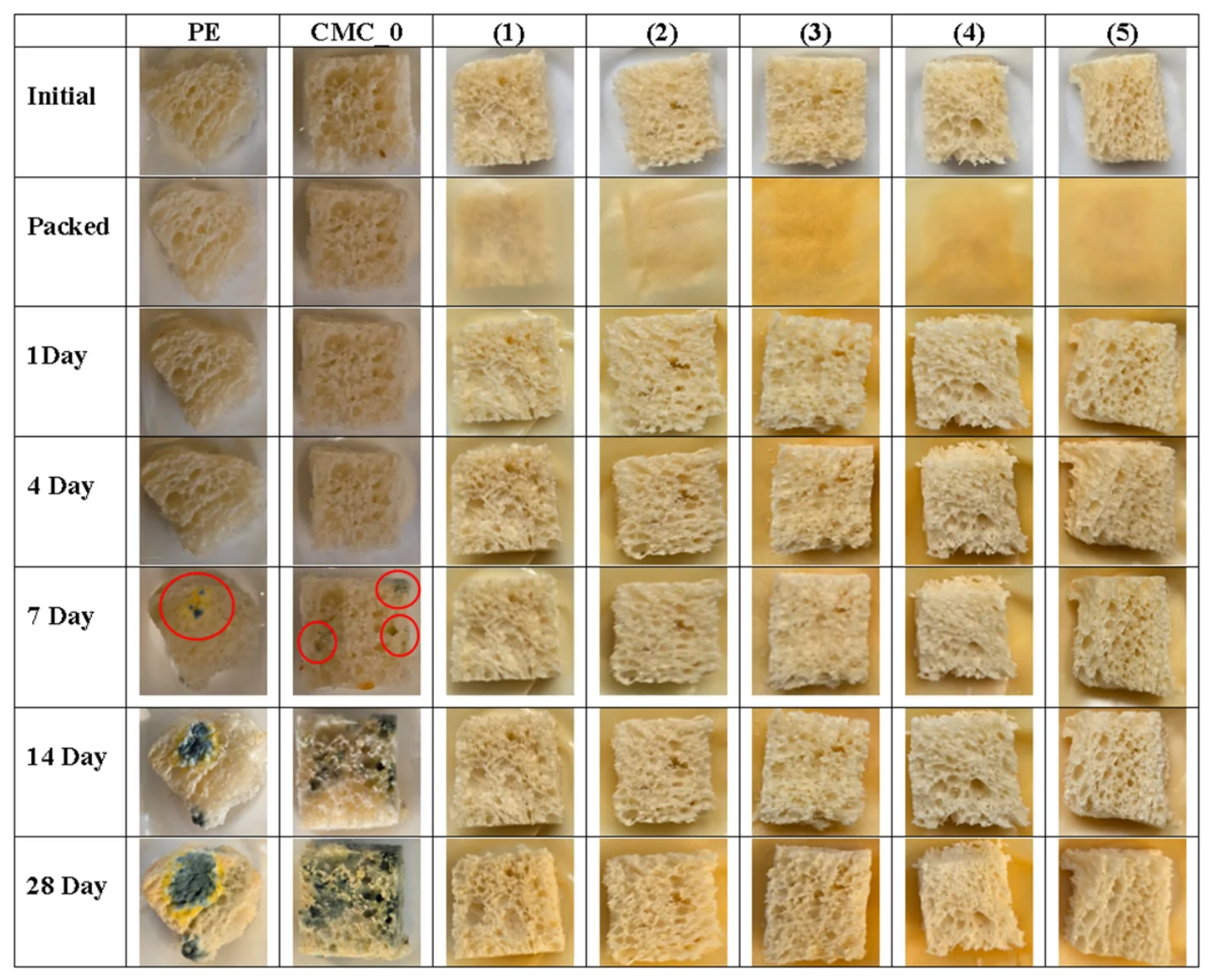
Visual appearance of bread bits wrapped in PE, CMC_0 control film and CMC_ZnO_CEO_1 (**1**)–CMC_ZnO_CEO_5 (**5**) composite films, initial and after 28 days’ storage at 25 °C and 30% relative humidity.

Visible mold growth was first observed on bread samples packaged in both CMC_0 and PE films after 7 days of storage at 25 °C. Mold development became more pronounced by day 14, and the samples exhibited extensive spoilage after 28 days of storage (Figure 9). In contrast, no visible mold growth was observed on any of the bread samples packaged in the composite films throughout the 28-day storage period, regardless of the ZnO NP or CEO content. These results demonstrate the high effectiveness of the developed composite films in delaying visible fungal growth and preserving the bread during the investigated storage period. The absence of visible mold growth over 28 days may also suggest sustained antimicrobial activity of the composite films. This behavior could be associated with the retention of CEO within the CMC matrix and its gradual release at the bread–package interface, thereby maintaining an antimicrobial environment over an extended period. Interactions between the CEO components, the CMC matrix and the ZnO NPs surface may contribute to the retention and controlled release of the active compounds. However, direct measurements of CEO release would be required to confirm this proposed mechanism [68]. The developed composite films therefore showed promising antimicrobial performance, effectively delaying visible fungal growth in the packaged bread samples. These findings suggest their potential application as active packaging materials for extending the shelf life of bread and other bakery products.

The potential application of cinnamaldehyde and cinnamon essential oil in bread packaging has previously been investigated using different packaging systems. Waxy paper incorporating 6% CEO was reported to almost completely inhibit the growth of *R. stolonifer* after 3 days [69]. Similarly, cinnamaldehyde incorporated into poly (butylene adipate-co-terephthalate) at concentrations of up to 10% was shown to effectively preserve bread [70]. In contrast, the incorporation of cinnamon powder into starch-based materials resulted in limited antimicrobial activity [71]. These findings highlight the importance of the chemical form of the cinnamon-derived active compound and its interaction with the polymeric packaging matrix in determining antimicrobial effectiveness.

### 3.9. Soil-Burial Test

**Figure 10:**
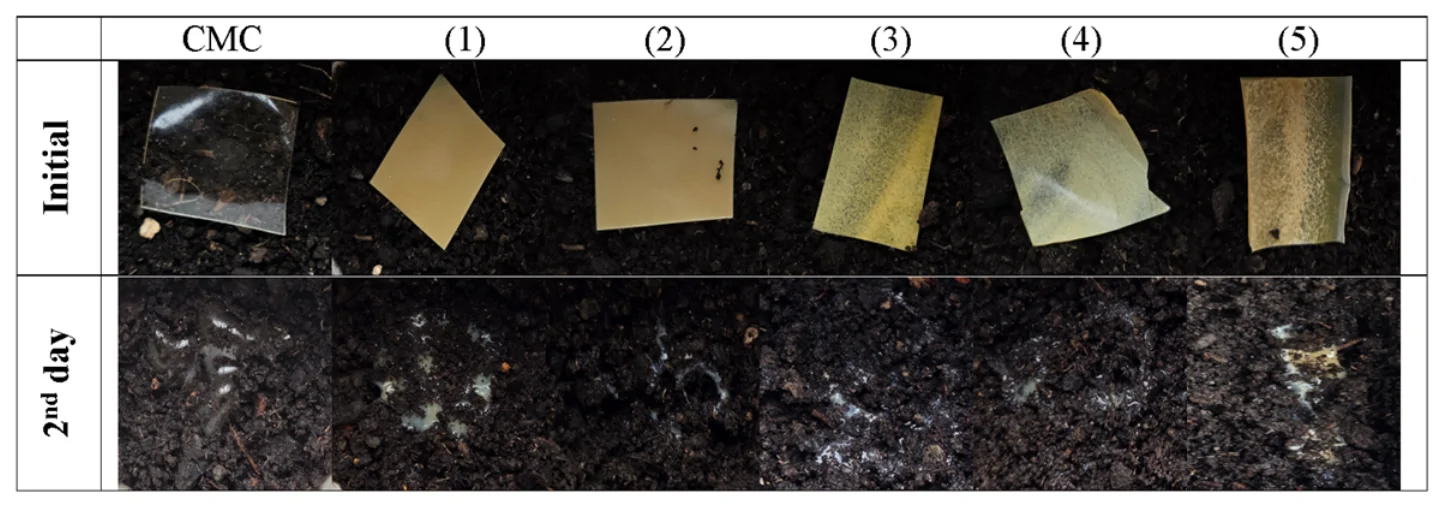
Visual disintegration of the CMC-based composite films from the 2nd day.

The degradation behavior of the cellulose-based composite films was evaluated under soil burial conditions. The samples were buried in soil and visually monitored at predetermined time intervals. Rapid deterioration of the composites was observed following burial, with substantial disintegration occurring during the first two days. By day 2, the samples were no longer visually detectable and could not be recovered from the soil (Figure 10).

The rapid disappearance of the composite during soil burial indicates a high susceptibility to disintegration, dissolution, and/or degradation under the tested soil conditions. Since these processes cannot be differentiated based solely on the observed disappearance of the material, further analyses are required to determine their respective contributions. Further analytical investigations would therefore be required to determine the relative contributions of physical disintegration, dissolution, and microbial degradation. The observed behavior may be associated with rapid moisture penetration into the hydrophilic CMC matrix, microbial activity, degradation of the polysaccharide matrix, and possible dissolution or transformation of the ZnO nanoparticles under the prevailing soil conditions [46]. In addition, microorganisms naturally present in soil may contribute to the degradation of the CMC matrix through enzymatic activity, as the polysaccharides represent an energy source [25].

## 4. Conclusions

Overall, the developed CMC/ZnO/CEO composite films demonstrate strong potential as sustainable active packaging materials for bakery products. The incorporation of ZnO NPs and CEO endowed the cellulose-based matrix with pronounced antimicrobial activity, while also improving its resistance to moisture transfer and maintaining sufficient optical transparency for product visibility. The antimicrobial effectiveness was demonstrated by inhibition zones of up to 38 mm and a reduction in planktonic bacterial viability below the detection limit after 4 h of contact. Furthermore, the successful preservation of bread without visible mold development during the 28-day storage period highlights the potential of these films for extending the shelf life of bakery products.

Importantly, the combination of antimicrobial activity, improved barrier performance and rapid disintegration under soil burial conditions provides a promising balance between food preservation functionality and end-of-life environmental performance. The rapid disappearance of the films under soil burial conditions suggests their susceptibility to environmental degradation; however, further studies based on standardized biodegradation methods and monitoring of degradation products are required to distinguish biodegradation from physical disintegration and dissolution. Overall, the findings support the potential of CMC/ZnO/CEO composites as an alternative to conventional packaging materials for applications where active protection against microbial spoilage and reduced environmental persistence are desired.

These findings also support the initial hypothesis that effective antimicrobial packaging films can be developed using components with established or intended food-related applications, reducing the need for potentially undesirable antimicrobial agents.

Nevertheless, some limitations of the present study should be acknowledged. The release behavior of CEO and ZnO from the active films was not investigated; therefore, the relationship between the release of these active components and the observed functional and antimicrobial properties could not be established. In addition, CMC-CEO and CMC-ZnO were not evaluated for all the properties investigated, limiting a comprehensive assessment of the individual contributions of CEO and ZnO and their comparison with the combined active films. Furthermore, a CMC-Tween 80 control film was not included, preventing the specific contribution of Tween 80 from being distinguished from that of CEO, particularly regarding CEO release and antibacterial activity. Although Tween 80 was used to facilitate the incorporation and dispersion of CEO within the CMC matrix, its potential influence on the observed results cannot be completely excluded. The absence of cytotoxicity and comprehensive safety assessments also represents a limitation, as the migration of active components and their potential effects under food-contact conditions were not evaluated. Future studies should therefore investigate the release and migration behavior of CEO and ZnO, include appropriate CMC-Tween 80 control films, systematically characterize CMC-CEO and CMC-ZnO across all relevant properties and assess cytotoxicity and overall food-contact safety before practical food-packaging applications can be fully assessed.

## Figures and Tables

**Table 1:** Codification and composition of cellulose-based composite films.

Sample Name (Code)	CMC	ZnO	CEO	Citric Acid	Glycerin	10% Tween 80 Sol. (% in Films)
CMC_0	5 g	-	-	2 g	2 mL	- (0%)
CMC_ZnO_CEO_1 (**1**)	5 g	0.25	1 mL	2 g	2 mL	10 mL (8.48%)
CMC_ZnO_CEO_2 (**2**)	5 g	0.25	2 mL	2 g	2 mL	10 mL (7.81%)
CMC_ZnO_CEO_3 (**3**)	5 g	0.25	3 mL	2 g	2 mL	10 mL (7.23%)
CMC_ZnO_CEO_4 (**4**)	5 g	0.15	2 mL	2 g	2 mL	10 mL (7.87%)
CMC_ZnO_CEO_5 (**5**)	5 g	0.50	2 mL	2 g	2 mL	10 mL (7.66%)

**Table 2:** Transmittance in UV and visible domains, opacity and thickness of the films.

Sample	Absorbance (A.U.)			Opacity	Thickness (mm)	Appearance
	UVA	UVB	A600			
CMC_0	0.118	0.364	0.0119	0.040 ± 0.000 ^a^	0.30 ± 0.00 ^a^	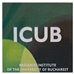
CMC_ZnO_CEO_1	1.215	1.136	0.0506	0.123 ± 0.003 ^b^	0.41 ± 0.01 ^b^	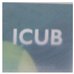
CMC_ZnO_CEO_2	1.211	1.132	0.0729	0.169 ± 0.007 ^c^	0.43 ± 0.02 ^b^	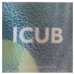
CMC_ZnO_CEO_3	1.183	1.111	0.0849	0.207 ± 0.010 ^d^	0.41 ± 0.02 ^b^	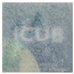
CMC_ZnO_CEO_4	1.211	1.129	0.0749	0.174 ± 0.004 ^c^	0.43 ± 0.01 ^b^	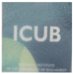
CMC_ZnO_CEO_5	1.203	1.117	0.0931	0.227 ± 0.016 ^d^	0.41 ± 0.03 ^b^	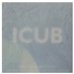

Different superscript letters within the same column indicate statistically significant differences between films (*p* < 0.05).

**Table 3:** The principal numerical data from thermal analysis.

Sample	T1% (°C)	T5% (°C)	T10% (°C)	Endo	Residual Mass (%)
CMC_0	59	116	170	93.8	18.83
CMC_ZnO_CEO_1	93	149	181	95.8	22.71
CMC_ZnO_CEO_2	91	139	179	105.2	23.35
CMC_ZnO_CEO_3	80	120	141	88.6/127.1	21.27
CMC_ZnO_CEO_4	63	115	151	95.0	20.98
CMC_ZnO_CEO_5	84	145	187	92.9	23.72

**Table 4:** Water vapor permeability values and mechanical properties for CMC0 and CMC_ZnO_CEO_1 (**1**)-CMC_ZnO_CEO_5 composite films.

Sample	WVP (10^−10^ g/Pa·m·s)	TS (MPa)	EB (%)	Young’s Modulus (MPa)
CMC_0	0.77 ± 0.02 ^a^	4.61 ± 0.15 ^a^	150.63 ± 16.61 ^a^	5.73 ± 0.75 ^a^
CMC_ZnO_CEO_1	0.65 ± 0.02 ^b^	7.07 ± 1.15 ^b^	56.82 ± 4.39 ^b^	23.82 ± 0.04 ^b^
CMC_ZnO_CEO_2	0.57 ± 0.01 ^c^	5.66 ± 1.37 ^a,b^	75.95 ± 16.93 ^b,c^	21.81 ± 8.59 ^b,c^
CMC_ZnO_CEO_3	0.45 ± 0.02 ^d^	4.29 ± 0.73 ^a^	77.95 ± 5.51 ^c^	19.33 ± 2.57 ^b^
CMC_ZnO_CEO_4	0.53 ± 0.03 ^c^	4.01 ± 0.67 ^a^	79.13 ± 13.40 ^b,c^	12.29 ± 1.34 ^c^
CMC_ZnO_CEO_5	0.46 ± 0.01 ^d^	8.61 ± 1.40 ^b^	59.20 ± 10.06 ^b^	61.05 ± 5.76 ^d^

Different superscript letters within the same column indicate statistically significant differences between films (*p* < 0.05).

## Data Availability

The original contributions presented in this study are included in the article/Supplementary Materials. Further inquiries can be directed to the corresponding author.

## Supplementary Materials

The following supporting information can be downloaded at: https://www.mdpi.com/article/10.3390/foods15203641/s1, Table S1: The obtained values for the diameter of the inhibition zone (mm); Table S2: The obtained values for the viability of bacterial cells (CFU/mL).
