# Supplementary material for: Enhanced Bread Shelf Life Using Carboxymethyl Cellulose Composite Films with Combined Antibacterial Activity of Zinc Oxide and Cinnamon Essential Oil
Source: Foods. 2026 Oct 14;15(20):3641. doi: 10.3390/foods15203641 (PMC13703537; doi:10.3390/foods15203641)
Supplement: Supplementary file 1 [file foods-15-03641-s001.zip › foods-4549186-supplementary.pdf]

Enhanced Bread Shelf Life Using Carboxymethyl Cellulose Composite Films with Combined Antibacterial Activity of Zinc Oxide and Cinnamon Essential Oil

Ludmila Motelica [1,2,3], Ovidiu-Cristian Oprea [3,4,5,*], Roxana Doina Trusca [3], Anton Ficai [3,4,5], George Pelin [6], Denisa Ficai [3,5], Carmen Curutiu [7]

[1] Research Institute of the University of Bucharest (ICUB), University of Bucharest, Spl. Independentei 91–95, 0500957 Bucharest, Romania; ludmila.motelica@upb.ro
[2] Research Center for Advanced Materials, Products and Processes, National University of Science and Technology POLITEHNICA Bucharest, Splaiul Independentei 313, 060042 Bucharest, Romania
[3] National Research Center for Micro and Nanomaterials, Faculty of Chemical Engineering and Biotechnologies, National University of Science and Technology POLITEHNICA Bucharest, Splaiul Independentei 313, 060042 Bucharest, Romania
[4] Academy of Romanian Scientists, Ilfov Street 3, 050044 Bucharest, Romania
[5] Faculty of Chemical Engineering and Biotechnologies, National University of Science and Technology POLITEHNICA Bucharest, Gh. Polizu 1–7, 011061 Bucharest, Romania
[6] INCAS—National Institute for Aerospace Research "Elie Carafoli", Bd. Iuliu Maniu 220, 061126 Bucharest, Romania
[7] Faculty of Biology, University of Bucharest, 030018 Bucharest, Romania; carmen.curutiu@bio.unibuc.ro
* Correspondence: ovidiu.oprea@upb.ro

Table S1. The obtained values for the diameter of the inhibition zone (mm)

Sample	Diameter of the inhibition zone (mm)	
	S. aureus ATCC 25923	*E. coli* ATCC 25922
CMC_0	6.0 ± 0.1^{a}	6.0 ± 0.1^{a}
ZnO	8.7 ± 0.6^{b}	7.7 ± 0.3^{b}
CEO	17 ± 0.6^{c}	16 ± 0.6^{c}
CMC_ZnO_CEO_1 (**1**)	25 ± 0.3^{d}	21 ± 0.3^{d}
CMC_ZnO_CEO_2 (**2**)	26 ± 0.6^{d}	24 ± 0.7^{e}
CMC_ZnO_CEO_3 (**3**)	38 ± 0.6^{e}	33 ± 0.6^{f}
CMC_ZnO_CEO_4 (**4**)	25 ± 0.4^{d}	24 ± 0.3^{e}
CMC_ZnO_CEO_5 (**5**)	30 ± 0.5^{f}	32 ± 0.5^{f}

Different superscript letters within the same column indicate statistically significant differences between films ($p < 0.05$).

Table S2. The obtained values for the viability of bacterial cells (CFU/mL)

Sample	Viability of bacterial cells 2h		Viability of bacterial cells 4h	
	S. aureus ATCC 25923	*E. coli* ATCC 25922	*S. aureus* ATCC 25923	*E. coli* ATCC 25922
Control	500,000 ± 20,000[a]	1,150,000 ± 10,000[a]	2,000,000 ± 20,000[a]	2,500,000 ± 20,000[a]
CMC_0	400,000 ± 25,000[b]	900,000 ± 25,000[b]	380,000 ± 20,000[b]	770,000 ± 15,000[b]
ZnO	290,000 ± 15,000[c]	600,000 ± 25,000[c]	BDL	165,000 ± 15,000[c]
CEO	275,000 ± 15,000[c]	135,000 ± 15,000[d]	BDL	BDL
CMC_ZnO_CEO_1 (**1**)	290,000 ± 20,000[c]	140,000 ± 20,000[d]	BDL	BDL
CMC_ZnO_CEO_2 (**2**)	255,000 ± 15,000[c]	130,000 ± 10,000[d]	BDL	BDL
CMC_ZnO_CEO_3 (**3**)	100,000 ± 5,000[d]	120,000 ± 15,000[d]	BDL	BDL
CMC_ZnO_CEO_4 (**4**)	290,000 ± 10,000[c]	160,000 ± 15,000[d]	BDL	BDL
CMC_ZnO_CEO_5 (**5**)	40,000 ± 15,000[e]	100 ± 100[e]	BDL	BDL

Different superscript letters within the same column indicate statistically significant differences between films ($p < 0.05$). BDL – below detection limit (< 100 CFU/mL)
